# Epigenetic Activation of *ASCT2* in the Hippocampus Contributes to Depression-Like Behavior by Regulating D-Serine in Mice

**DOI:** 10.3389/fnmol.2017.00139

**Published:** 2017-05-09

**Authors:** Jiesi Wang, Ke Zhang, Xiaojuan Chen, Xiaoqian Liu, Huajing Teng, Mei Zhao, Zhongsheng Sun

**Affiliations:** ^1^Beijing Institutes of Life Science, Chinese Academy of SciencesBeijing, China; ^2^Key Lab of Mental Health, Institute of Psychology, Chinese Academy of SciencesBeijing, China; ^3^University of Chinese Academy of SciencesBeijing, China; ^4^Institute of Genomic Medicine, Wenzhou Medical CollegeWenzhou, China

**Keywords:** ASCT2, SLC1A5, D-serine, depression, chronic social defeat stress, mouse

## Abstract

The roles of D-serine in depression are raised concerned recently as an intrinsic co-agonist for the NMDA receptor. However, the mechanisms underlying its regulation are not fully elucidated. ASCT2 is a Na^+^-dependent D-serine transporter. We found that decreased D-serine and increased hippocampal ASCT2 levels correlated with chronic social defeat stress (CSDS) in mice. Lentivirus-mediated shRNA-mediated knockdown of ASCT2 and the administration of exogenous D-serine in the hippocampus alleviated CSDS-induced social avoidance and immobility. *In vivo* and *in vitro* experiments revealed that upregulation of *ASCT2* expression in CSDS was regulated through histone hyper-acetylation, not DNA methylation in its promoter region. Immunohistochemistry demonstrated the co-localization of ASCT2 and D-serine. Uptake of D-serine by ASCT2 was demonstrated by *in vivo* and *in vitro* experiments. Our results indicate that CSDS induces ASCT2 expression through epigenetic activation and decreases hippocampal D-serine levels, leading to social avoidance, and immobility. Thus, targeting D-serine transport represents an attractive new strategy for treating depression.

## Introduction

Depression, a severe chronic mental illness, is a leading cause of disability worldwide. Although monoamine deficiency has been suggested as a major mechanism underlying depression pathogenesis, and the monoamine hypothesis has dominated the development of pharmacological treatments for depression, monoamine-based drugs provide relief for less than half of the patients diagnosed with major depressive disorder. Moreover, delayed response to monoamine antidepressants is commonly observed in clinical practice (Morilak and Frazer, 2004). Thus, rapidly acting antidepressants are considered an attractive approach for treating depression.

Glutamate is a major excitatory neurotransmitter, which mediates the vast majority of fast excitatory transmission in the brain (Baker et al., 1991). Several lines of evidence indicate the involvement of ionotropic glutamate receptors, including the α-amino-3-hydroxy-5-methyl-4-isoxazolepropionic acid (AMPA) receptor and N-methyl-D-aspartate (NMDA) receptor, in pathological mechanisms underlying depression (Kim et al., 1982; Levine et al., 2000; Yuksel and Ongur, 2010; Jiang et al., 2013). Recent preclinical and clinical studies have reported a rapid anti-depressive effect with low doses of high-affinity non-competitive NMDA receptor antagonists, including dissociative anesthetic ketamine (Li et al., 2010, 2011). Multiple case reports indicate at least 60% response rate to ketamine injection in patients with treatment-resistant depression, and this response is acute and sustained. However, the antidepressant mechanism of ketamine has not been elucidated; its antidepressant effect may be partially due to its antagonism of NMDA receptors, while it has also been reported that ketamine works as a rapid neuroplastic modulator (Serafini et al., 2014). These investigations into ketamine provided novel and intriguing insight into depression treatment, and glutamatergic drugs targeting NMDA receptors present a new direction for the development of rapid-acting antidepressants.

NMDA receptor activation depends on the simultaneous binding of glutamate and a co-agonist, such as glycine and D-serine (Mathews et al., 2012). Compared to L-glycine,D-serine is considered a more potent NMDA receptor co-activator and is involved in the modulation of synaptic activity and plasticity (Yang et al., 2003; Panatier et al., 2006), neuronal migration (Schell et al., 1997; Kim et al., 2005), and excitotoxicity (Katsuki et al., 2004; Shleper et al., 2005). Recent studies have reported that the Flinders sensitive line (FSL) rat, a model of depression, revealed lower levels of D-serine in the hippocampus (Gomez-Galan et al., 2013), while D-serine administration alleviated depression-like behavior as evidenced by decreased immobility in the forced swim test (FST) and learned helplessness in rodents (Malkesman et al., 2011, 2012; Otte et al., 2013). In addition, transgenic SrrTg mice expressing serine racemase (SRR) which converts L-serine to D-serine, show reduced proneness toward depression-like behavior, while chronic dietary D-serine supplementation mimics the depression-resistant phenotype observed in SrrTg mice (Otte et al., 2013). However, the mechanisms underlying D-serine regulation in depression have not been clarified.

D-serine is converted from L-serine by SRR and degraded by D-amino acid oxidase (DAAO; Nishikawa, 2011). Physiological pathways controlling D-serine transport play a role in the regulation of extracellular D-serine concentration in the central nervous system (CNS). An integral membrane amino acid transporter, ASCT2 (also known as ATBO or SLC1A5) is a member of the ASC family of Na^+^-dependent substrate exchangers, expressed in neurons and astrocytes (Kanai and Hediger, 2003). D-serine uptake by ASCT2 has been reported in cultured cortical neurons and C6 glioma cells (Shao et al., 2009; Sikka et al., 2010). In addition, ASCT2-like low-affinity Na^+^-dependent D-serine uptake in P2 synaptosomal membrane fractions has also been observed (Gliddon et al., 2009). Wolosker et al. have suggested a model of D-serine metabolism, called the glia-neuron serine shuttle: astrocytes synthesize and export L-serine to neurons, where it is converted by neuronal SRR to D-serine, which is then uptaken to storage, metabolism, and activity-dependent release (Wolosker, 2011; Wolosker and Radzishevsky, 2013).

Recent studies have identified epigenetic mechanisms, such as histone modification and DNA methylation, as important factors mediating the effect of external stress on gene expression, and ultimately influencing long-lasting behavioral responses (Fass et al., 2014). Existing evidence suggests that changes in the DNA methylation level of specific genes are associated with vulnerability to depression (Hunter et al., 2009; Murgatroyd et al., 2009; Wilkinson et al., 2009; Zhang et al., 2010; Bagot et al., 2012). Chronic social defeat stress (CSDS) upregulated histone H3 acetylation in the rat hippocampus (Hollis et al., 2010), while long-term treatment with imipramine increased NR2B expression in mouse cortical neurons, via epigenetic changes, including increased histone H3K9 and H3K27 acetylation in the NR2B promoter (Nghia et al., 2015). These findings indicate that epigenetic modifications play an important role in the pathophysiology and treatment of depression (Bagot et al., 2014).

In mice, CSDS induces depression-like behavior, including increased immobility in the FST and social avoidance and is therefore becoming an increasingly popular model of depression (Berton et al., 2006; Sun et al., 2012; Donahue et al., 2014). In this study, we used this model to investigate if ASCT2-regulated D-serine transport is involved in the development of depression and the underlying epigenetic mechanisms of ASCT2 regulation.

## Materials and methods

### Animals

We used C57BL/6J male mice (Vital River, Beijing, China) that were 10 weeks old at the start of all the experiments. All experiments were performed in strict accordance with the Guidelines for Care and Use of Laboratory Animals of the Chinese Academy of Sciences. All protocols were approved by the Review Board of the Institute of Psychology, Chinese Academy of Sciences (protocol number: A12031, May 2012). In the present study, eight batches of mice were used. The first batch of mice (*n* = 6 for each group) was used in the total hippocampal D-serine analysis, the second (*n* = 4–5 for each group) in the microdialysis experiment, the third (*n* = 5 for each group) in the real-time PCR for gene *ASCT2*, the fourth batch (*n* = 7–8 for each group) was used in the real-time PCR for genes *DAAO* and *Srr* and the Western blot and DNA methylation analysis, the fifth (*n* = 7–8 for each group) in immunostaining of ASCT2 and D-serine, the sixth (*n* = 10–12 for each group) and seventh (*n* = 7–10 for each group) in D-serine administration and lentivirus infection, respectively; and the eighth (*n* = 6 for each group) in the CHIP analysis.

### Intra-hippocampal infusion of lentivirus

The surgery for lentivirus administration was performed 24 h after the last social stress test. Briefly, the animals were isoflurane-anesthetized and placed into a stereotaxic apparatus (Kopf instruments, Tujunga, CA). Burr holes were drilled, a Hamilton syringe needle was lowered, and the lentivirus was infused bilaterally at a rate of 0.1 μL per min to a total volume of 0.5 μL. The coordinates for the needle tip as measured from Bregma were as follows: anteroposterior −1.75 mm, lateral ± 2.4 mm, and dura −2.6 mm. The needle remained in place for 10 min following the injection to limit suction of the lentivirus up the needle track. Mice were euthanized 14 days after viral injection. Four to five mice of each group were perfused with 4% paraformaldehyde, and brain slices were immunostained with the anti-GFP antibody to locate the infection region and check its efficiency. Five mice of each group were euthanized to measure D-serine concentration in the CA3 region, which was isolated from 1-mm-thick brain coronal slices contained in this area.

### Intra-hippocampal D-serine administration

The surgery for D-serine administration was performed 7 days prior to the social interaction test. Mice were placed into a stereotaxic apparatus and bilateral guide cannulae (OD 0.46 mm, Plastics One, Roanoke, VA) were surgically implanted targeting the hippocampus similar to lentivirus infusion; the same coordinates were used and the cannulae were permanently fixed to the skull with Loctite skull adhesive (Henkel, Rocky Hill, CT). D-serine infusion was performed using a microinjection pump 20 min prior to behavioral testing. Twenty-four hours after the last behavioral test, the mice received intra-core Cresyl Fast Violet; 30 min later, they were anesthetized and perfused with phosphate buffered saline (PBS) and 4% paraformaldehyde. Nissl staining of the brain slices was performed using the standard protocol to confirm the site of drug injection.

### *In vivo* microdialysis

Mice were anesthetized and surgically implanted with a Guide Cannula aimed at the right hippocampus 7 days prior to the social stress test. The coordinates for the needle tip as measured from Bregma were as follows: anteroposterior—2.3 mm, lateral—1.2 mm, dura—1.2 mm with a 24° angle relative to the sagittal plane. After testing social interaction behavior, hippocampal microdialysis was performed. Microdialysis probes (6000 Dalton, 1 mm length, OD 0.24 mm, CMA, Kista, Sweden) were sequentially submerged in ethanol and saline for 5 min in each, concurrently with being perfused by a flowed compound sodium chloride solution to (5.0 μL/min). Mice were briefly anesthetized with isoflurane and fitted with a plastic collar, and then probes were inserted into the hippocampus through the guide cannula. Flow rate of syringe pumps (CMA, MA) were set to 1.0 μL/min. Dialysate samples were subsequently collected every 15 min for 1.5 h using a refrigerated fraction collector (Univentor 820 Microsampler, Zejtun, Malta) and the samples collected in the first 0.5 h were discarded. Mice were awoken and let to move freely in a specialized cage (CMA, MA) during collection of dialysate samples.

### Chronic social defeat stress (CSDS)

CSDS was performed using a procedure similar to that reported previously (Berton et al., 2006; Tsankova et al., 2006). Briefly, for CSDS group, C57 mice were put in contact with a different CD-1aggressor for 5 min each day over a total of 10 days. Every day, after the 5 min confrontation, the test and CD-1 mice were separated into adjacent compartments divided by a plastic wall containing holes in the middle, which provided chronic exposure the aggressor for the next 24 h. Control mice were housed in equivalent cages but with animals of the same strain without directly interaction. Twenty-four hours after the last session, all mice were housed individually for 10 days and then examined with the social interaction test or FST. Tissues of mice which showed social avoidance behavior were collected and subjected to qPCR, Western blot analysis, and immunohistochemistry.

### Social interaction test

The social interaction test was performed similarly to previous studies (Berton et al., 2006; Tsankova et al., 2006). During the social interaction test, a stressed or control C57 mouse was placed in an open field (42 × 42 cm) with a small empty metal cage (5 × 10 cm). An 8 cm zone around the small cage was considered as an interaction zone, and the time the mouse spent there over 2.5 min was recorded automatically by the Video Detecting System (Anilab, Ningbo, China). Then, a CD-1 aggressor mouse was introduced into the cage and the procedure was repeated. The interaction score was calculated as the time the C57 mouse spent in the interaction zone in the presence of the aggressor divided by the time spent there in the absence of the aggressor.

### Forced swim test

Mice were transferred to the experimental room and allowed to acclimate for 1 h. Thereafter, they were placed for 6 min into a glass cylinder filled with water (20 cm high, 25°C; Porsolt et al., 1977). The mice were judged immobile when they ceased struggling and remained floating motionless in the water (without any vertical or horizontal movements), performing only the movements necessary to keep their heads above the water level. To control for the effect of the FST on the molecular test that followed, all tissues were collected from mice that were not subjected to the test.

### RNA, DNA, and protein isolation

For tissue samples, total RNA, DNA, and protein were isolated using AllPrep DNA/RNA/Protein Mini Kit (QIAGEN, Dusseldorf, Germany) according to the manufacturer's instructions. Proteins were dissolved in 1% sodium dodecyl sulfate, and their concentration was measured using the BCA assay (Biyotime, Nantong, China). For cell samples, total RNA was isolated using Trizol reagent (Life Technologies, Carlsbad, CA). The quality of isolated RNA and DNA was determined by agarose gel electrophoresis using 1% non-denaturing gels, and the concentration was measured using Nano-drop 2000 c spectrometer (Thermo Fisher Scientific, Waltham, MA).

### Reverse transcription and real-time PCR

Total RNA (2 μg) was utilized for the first-strand cDNA synthesis using the M-MLV Protoscript kit (NEB, Ipswich, MA) according to the manufacturer's protocol. Expression levels were determined by real-time quantitative qPCR using SYBR Green Mix (CWBIO, Beijing, China). PCR conditions were: 95°C for 10 min, 40 cycles at 95°C for 10 s, 60°C for 60 s, and signal detection for 10 s; dissociation curve analysis was performed at the end of each run. Primers for qPCR were designed using the Perlprimer software. Each amplification reaction was performed in duplicate unless noted otherwise, and relative mRNA expression was determined by the standard ΔΔCt method using glyceraldehyde-3-phosphate dehydrogenase (*GAPDH*) as a normalization control.

### Bisulfite sequencing and DNA methylation analysis

To convert non-methylated cytosine to uracil leaving 5-methylcytosines unmodified, 500 ng of DNA samples were treated using the EZ DNA Methylation-DirectTM Kit (Zymo Research, Irvine, CA) according to the manufacturer's protocol; and utilized as a template for nested PCR to amplify the region within the *ASCT2* promoter. PCR products were gel-purified and inserted into the pGEM-T vector (Promega, Beijing, China). The transformed clones (at least 20 from each sample) were used to purify the plasmids, which were sequenced in the Beijing Genomics Institute (BGI, Beijing, China).

### Quantitative chromatin immunoprecipitation (ChIP) assay

Hippocampal tissues were treated using the Magna CHIPTM G Tissue kit (Millipore, Darmstadt, Germany) according to the manufacturer's protocol and sonicated using a Biorupter Pico instrument (Diageuode, Japan) for 12.5 min (45 s, on; 30 s, off; 10 cycles). Cell samples were treated using the Zymo-Spin™ ChIP Kit (ZymoResearch, Irvine, CA) according to the manufacturer's protocol and sonicated for 10 min (30 s, on; 30 s, off; 10 cycles). Cross-linked chromatin was immunoprecipitated with antibodies against acetylated chromatin: anti-H3K9 (ab10812; Abcam, Hong Kong, China), anti-H3K27 (ab3350; Abcam), and anti-H3k4 (#ABE223; Millipore); normal rabbit IgG-B (sc-2763; Santa Cruz Biotechnology, Dallas, TX) was used as a negative control antibody. Three pairs of primers specific for the *Asct2* promoter region (see **Figure 4G**) were used to detect the acetylation status of H3K9, H3K4, and H3K27. Real-time PCR was performed in triplicate.

### Western blot analysis

Total proteins were separated by sodium dodecyl sulfate-polyacrylamide gel electrophoresis (SDS-PAGE) in reducing conditions using 10% acrylamide gels and analyzed by immunoblotting using anti-ASCT2 (1:500; ab84903, Abcam), anti-SRR (1:500; ab45434, Abcam), anti-GAPDH (1:5,000; #5174, CST, Danvers, MA) and anti-beta-actin (1:2,000, #4970, CST) as primary antibodies; anti-rabbit HRP-conjugated IgG (1:5,000, #7074, CST) was used as a secondary antibody. Band intensity was semi-quantitatively analyzed using the Quantity One software (BioRad, Hercules, CA), and the expression level of ASCT2 was normalized to that of GAPDH.

### Immunohistochemistry

Mice were anesthetized by chloral hydrate and then perfused with 0.1 M PB, pH 7.4, containing 4% paraformaldehyde, 0.1% glutaraldehyde, and 1% sodium metabisulfite for immunostaining of D-serine (Panatier et al., 2006) or only 4% paraformaldehyde for other experiments. The brains were removed and immersed in the same fixative for 2 h at room temperature. The sections were sliced from the hippocampal area (−1.7 to −2.7 to Bregma), and three sections (40 μm for each section) were selected (section1 located in −1.7 to −1.9; section2 located in −2.1 to −2.3; section3 located in −2.5 to −2.7). Sections were blocked in PBS containing 10% serum and 1% Triton X-100 for 1 h at room temperature. For single immunostaining, sections were incubated with rabbit anti-ASCT2 (1:200; Abcam) or rabbit anti-GFP (1:1,000; Santa Cruz Biotechnology) antibodies for 24 h at 4°C and then with goat anti-rabbit FITC- or Alexa Fluor® 555-labeled secondary antibodies (1:1,000; Life Technologies). For double immunostaining, sections were incubated with a mixture of rabbit anti-D-serine (1:1,000; Abcam) and goat anti-ASCT2 (1:200; Santa Cruz Biotechnology) for 48 h at 4°C, and then with donkey anti-rabbit Alexa Fluor® 488-labeled and donkey anti-goat Alexa Fluor® 594-labeled secondary antibodies (1:1,000; ab150080 and ab150077, respectively; Abcam). The Anti-D-serine antibody had a 1/ >50,000 cross reactivity ratio to L-serine, and its specificity to D-serine was confirmed by comparing brain regions where it was highly and rarely expressed. All antibodies were diluted in PBS containing 2% serum and 0.5% Triton X-100. Three hippocampal sections from each mouse were stained, and the images were analyzed using a confocal scanning microscope (Olympus, LV1200, Shanghai, China). Fluorescence intensity in the pyramidal cell layer was analyzed using the ImageJ software, and the average of three sections' mean gray value was calculated as the intensity of each sample.

### High-performance liquid chromatography (HPLC)

HPLC was performed with some modifications described in previous studies (Grant et al., 2006; Gomez-Galan et al., 2013). Every whole hippocampus or CA3 region was homogenized, sonicated in 500 or 150 μL of 0.1 M NHCLO_4_, respectively, and centrifuged to remove any insoluble matter. Each sample or standard solution was derivatized with the same volume of o-phthalaldehyde (OPA)/N-isobutyryl-l-cysteine (IBC) (Sigma, St. Louis, MA) mixture (1 mg OPA and 2 mg IBC in 0.1 mL methanol, followed by 0.9 mL of 0.2 M sodium borate buffer, pH 10.0). Amino acid separation was performed on a Waters CORTECS C18 + column (4.6 × 150 mm, 2.7 μm) by a liner gradient elution. Mobile phase A contained 850 mL 0.04 M sodium phosphate and 150 mL methanol, and mobile phase B contained 670 mL 0.04 M sodium phosphate, 555 mL methanol, and 30 mL tetrahydrofuran, pH 6.2 (Grant et al., 2006); the applied linear gradient was 15–25% B for 40 min, 25–100% B for 5 min, and 100 to 15% B for 10 min, then maintained for 15 min; the flow rate was 0.4 ml/min. The total run time was 70 min. The samples were analyzed by measuring fluorescence (the excitation and emission wavelengths of 340 and 450 nm, respectively) using an Agilent 1100 Series FLD G1321A Fluorescence Detector (Agilent, Santa Clara, CA).

### Cell culture

HEK293T or HT22 cells were cultured at 37°C and 5% CO_2_, and the culture medium, unless noted otherwise, was Dulbecco's modified Eagle's medium (DMEM) supplemented with 10% fetal bovine serum (FBS), 1% Penicillin/Streptomycin (HyClone, Beijing, China).

### Lentivirus packaging

Six lentiviral vectors [3 expressing *Asct2*-specific shRNA, 1 expressing scrambled (SCR)-shRNA, 1 expressing *Asct2*] under the control of the mU6 promoter and EGFP under the control of the CMV promoter were purchased from Applied Biological Materials (Vancouver, BC). Lentiviral vectors were packaged in accordance with the manufacturer's protocol. Viral particles were purified from the medium, concentrated using the ViraTrap Lentivirus Purification Miniprep Kit (Biomiga, San Diego, CA) and diluted in PBS to a final concentration of 1 × 10^12^ viral genomes per mL (VG/mL). Viral titer was determined as 1 × 10^7–8^ transducing units per ml (TU/ml) by serial dilution and plate counting using HT22 hippocampal cellsASCT2 expression was determined by qPCR in HT22 cells infected by lentiviruses expressing *Asct2*, SCR-shRNA or *Asct2*-specific shRNAs, respectively (**Figures 3A,B**); the *Asct2*-specific shRNA lentivirus producing the highest inhibition of ASCT2 expression (Lenti-1127) was selected for mouse injection and cell infection.

### D-serine uptake in infected HT22 cells

HT22 cells were plated into 3 wells of a 6-well plate and infected by lentiviruses which express *Asct2* (overexpression), *Asct2*-specific shRNA (Lenti-1127) (downregulation) or scramble shRNA (control), respectively. After 48 h of infection, cells were incubated in a medium containing 2 μg/ml of puromycin for 72 h when only successfully infected (GFP positive) cells were alive. Further, cells were incubated in a medium containing 1 μg/ml puromycin to expand the culture. To determine the D-serine uptake, cells were plated in a 96-well-plate (5 × 10^4^/well, 3 wells for each infected cell line) to culture for 24 h, and then the medium was changed to 0.1 ml complete medium containing 7 μM D-serine for another 24 h. After that, the medium was collected for HPLC examination, and the rest of the cells were used to measure cell metabolic activity with a MTT assay kit (Solarbio, Beijing, China) following the manufacturer's protocol. The uptake of amino acids was calculated as follows:

$$\begin{array}{l} \text{Amino acids uptake}=(\text{the initial concentration}\\ \;-\text{ the remaining concentration})\times \\ \text{ volume }\times \text{ the relative metabolic activity} \end{array}$$

The activity of the control was defined as 100%.

### HDAC inhibitor administration to HT22 cells

HT22 cells were used to test Lentivirus efficiency or administrated with suberoylanilide hydroxamic acid (SAHA), a HDAC inhibitor. The SAHA was dissolved in DMSO and added into the medium to final concentrations (5 or 10 μM). After 6 h incubation, the cells were collected to use for CHIP assay or qPCR.

### Data analysis

Data are expressed as the mean ± S.E.M. Statistical analyses were performed with the GraphPad Prism 6.0 software. Means between two groups were compared with two-tailed Student's *t*-tests. We performed *F*-test prior to conducting *t*-tests, and found no significant results. Comparisons of means of multiple groups were analyzed with two-way ANOVA followed by Fisher's LSD *post-hoc* tests. Methylation levels at different CG sites were analyzed online at http://quma.cdb.riken.jp/. Statistical significance was set at *p* < 0.05.

## Results

### Hippocampal D-serine contributes to depression-like behavior induced by chronic social defeat stress (CSDS)

**Figure 1:**
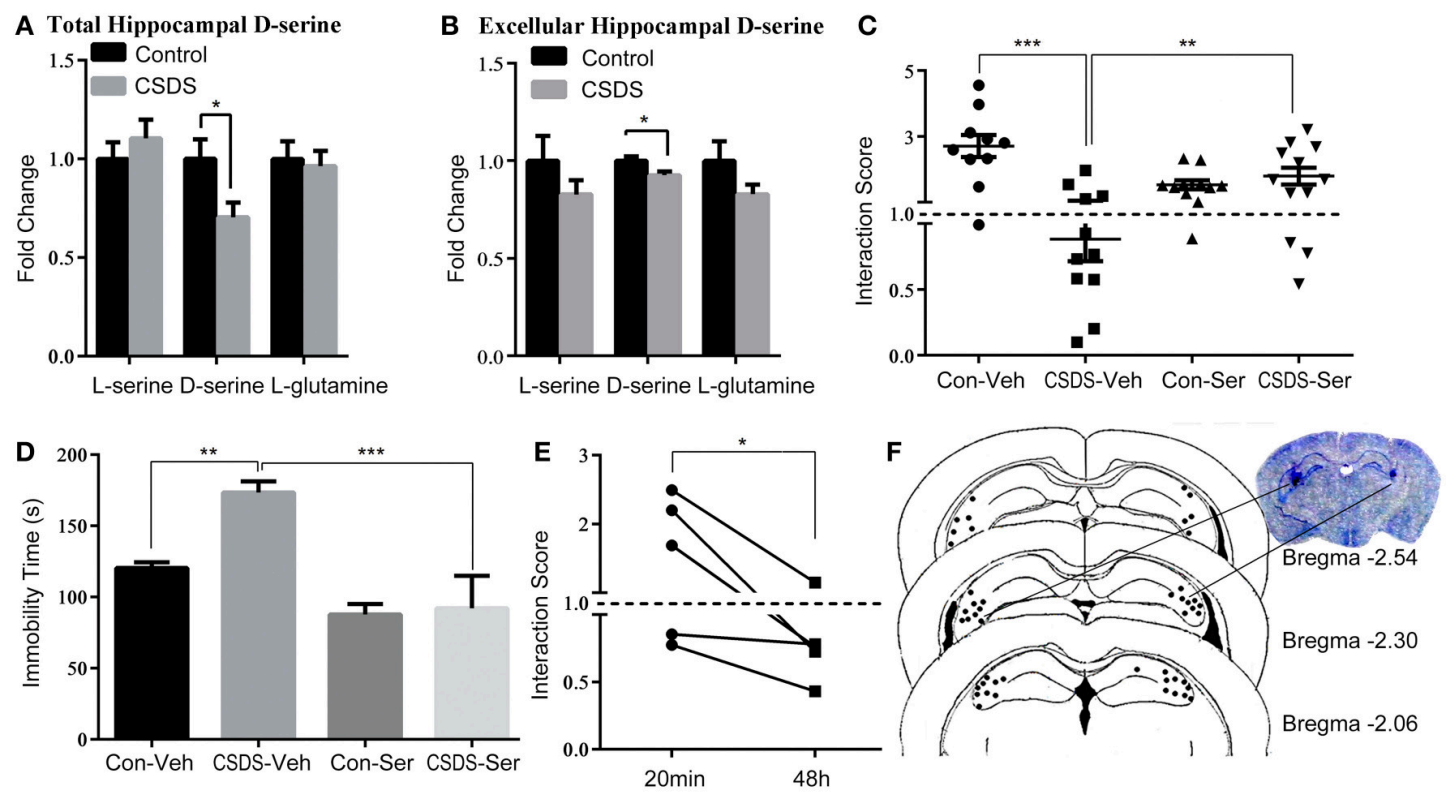
**D-serine levels in the hippocampus define depression-like behavior in response to chronic social defeat stress (CSDS) in mice. (A)** Relative levels of L-serine, D-serine, and L-glutamine in the hippocampus after CSDS (*n* = 6, ^*^p < 0.05, control vs. CSDS mice; unpaired *t*-test). **(B)** Relative levels of L-serine, D-serine, and L-glutamine of hippocampal intercellular fluid after CSDS (*n* = 4–5, ^*^p < 0.05, control vs. CSDS mice; unpaired *t*-test) **(C)** Social interaction score and **(D)** immobility time in the forced swim tests (FST) after acute D-serine administration (20 min prior to the test) into the hippocampus (*n* = 10–12; ^*^p < 0.05, ^**^p < 0.01, ^***^p < 0.001; two-way ANOVA). **(E)** Re-testing of social interaction 48 h after D-serine administration (*n* = 5, ^*^p < 0.05, 20 min vs. 48 h; paired *t*-test). **(F)** The location of injection sites in the hippocampus.

To determine the role of hippocampal D-serine in depression-like behavior, both the level of D-serine of hippocampal tissue and intercellular fluid, which were collected by microdialysis, were measured using high performance liquid chromatography (HPLC). Our analysis indicated that D-serine was significantly decreased in the hippocampus of CSDS mice (unpaired *t*-test, *p* < 0.05; Figures 1A,B), whereas L-serine and L-glutamine were not significantly changed. Further, we administered D-serine directly into the hippocampus 20 min prior to the social interaction test and examined social interaction score (SIS) and immobility in the FST for stressed and unstressed mice. The results of the social interaction test showed a significant main effect of stress [*F*_(1, 40)_ = 11.31, *p* < 0.01] and interaction effect of stress × D-serine [*F*_(1, 40)_ = 20.27, *p* < 0.001], but no D-serine effect [D-serine *F*_(1, 40)_ = 0.3228, *p* > 0.05]; *Post-hoc* analysis showed that in the groups with vehicle administration, the SIS was significantly lower in CSDS mice (CSDS-Veh vs. Con-Veh; *p* < 0.001), indicating that CSDS induced robust and lasting social avoidance behavior. After D-serine administration, SIS was significantly higher in D-serine treated mice (CSDS-Ser vs. CSDS-Veh; *p* < 0.01; Figure 1C). Corresponding results were obtained in the FST, two-way ANOVA revealed a significant main effect of stress [*F*_(1, 17)_ = 5.499, *p* < 0.05] and D-serine [*F*_(1, 17)_ = 21.77, *p* < 0.01], and also observed a marginally significant interaction effect [*F*_(1, 17)_ = 4.004, *p* = 0.062]. *Post-hoc* analysis revealed that CSDS mice exhibited increased immobility compared to control mice (Con-Veh vs. CSDS-Veh; *p* < 0.01), and D-serine treatment decreased the immobility compare to vehicle treatment (CSDS-Veh vs. CSDS-Ser; *p* < 0.001; Figure 1D). However, social avoidance behavior reappeared 48 h after D-serine administration (20 min vs. 48 h after treatment, paired *t*-test, *p* < 0.05), indicating that the anti-depressive effect of D-serine was rapid but transient (Figure 1E). Figure 1F shows the delivery site of D-serine checked by Nissl staining.

### Regulation of ASCT2 in the hippocampus by CSDS

**Figure 2:**
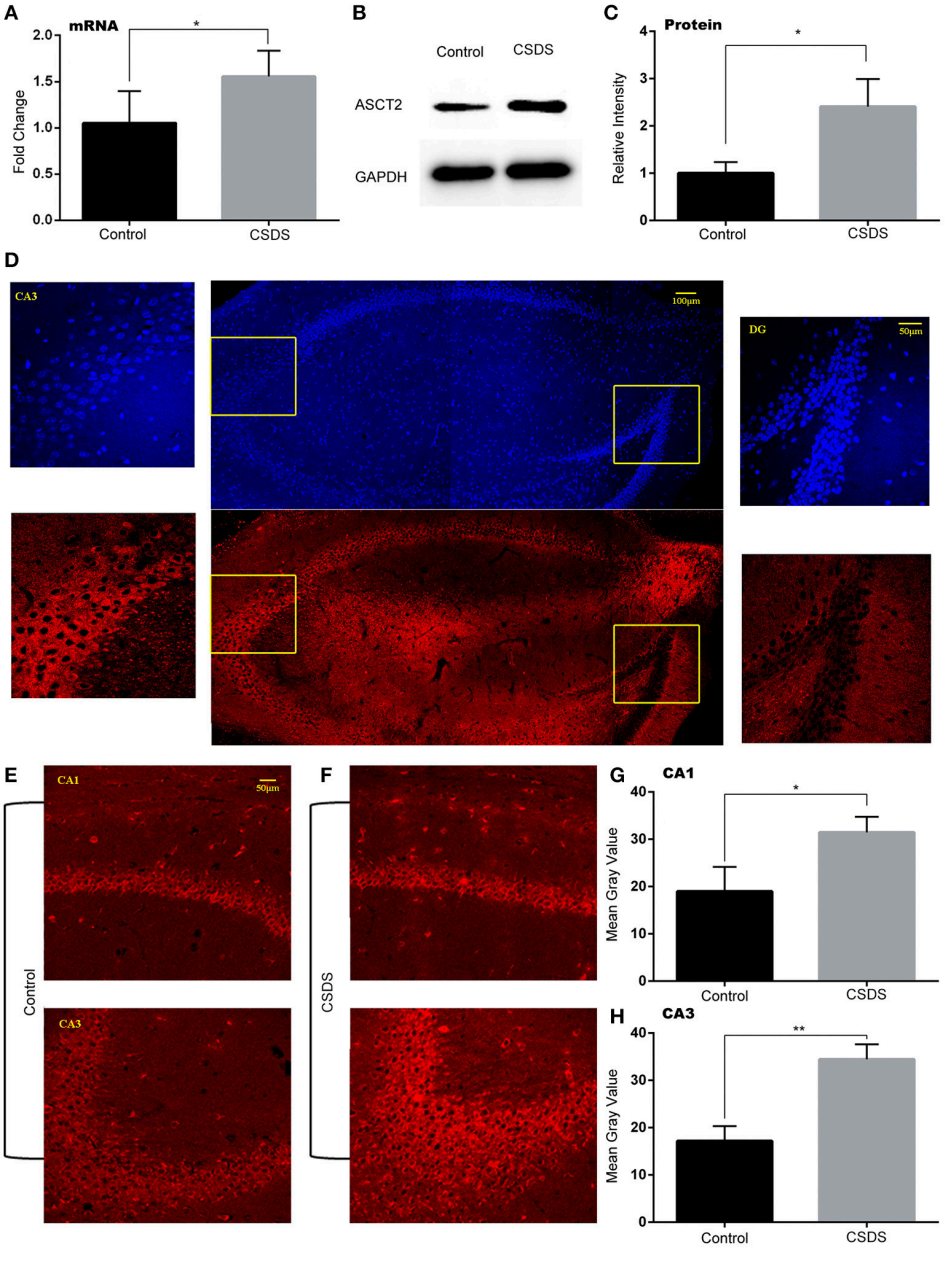
**Increased ASCT2 expression in the mouse hippocampus after CSDS**. **(A)** Relative *Asct2* mRNA expression (*n* = 5, ^*^p < 0.05, CSDS vs. control mice; unpaired *t*-test). **(B,C)** Western blot analysis of ASCT2; representative immunoblot **(B)**; quantification of ASCT2 protein expression **(C)** (*n* = 7, ^*^p < 0.05, CSDS vs. control mice; unpaired *t*-test). **(D)** The distribution of ASCT2 in the hippocampus presented by immunostaining. **(E,F)** Immunostaining of ASCT2 in the CA1 (upper image) and CA3 (lower image) regions of the hippocampus in control **(E)** and CSDS **(F)** mice. **(G,H)** Quantification of ASCT2-specific immunostaining (*n* = 5). The data are expressed as mean ± S.E.M. of the mean gray value (^*^p < 0.05 for CA1 and ^**^p < 0.01 for CA3, CSDS vs. control mice; unpaired *t*-test).

The role of ASCT2, an important D-serine transporter, in mood disorders has not been yet investigated. To determine whether ASCT2 plays a role in depression-like behavior, we measured ASCT2 expression in the hippocampus of CSDS mice by quantitative qPCR and Western blot analysis. The results showed that hippocampal ASCT2 expression was significantly increased at both mRNA (+50.3%; *p* < 0.05. Figure 2A) and protein levels (+141%; *p* < 0.05; Figures 2B,C) in CSDS mice. Further, we examined the distribution of the ASCT2 protein in the hippocampus by immunohistochemistry. The results revealed that ASCT2 was mostly localized in the cornus ammonis areas CA1 and CA3, while being scarcely observed in the dentate gyrus (DG) (Figure 2D). In addition, fluorescence intensity analysis showed that ASCT2 expression was significantly increased in CA1 (Control vs. CSDS mice, *p* < 0.05, Figures 2D–F) and CA3 in CSDS mice (Control vs. CSDS mice, *p* < 0.01, Figures 2D,E,G,H). These results suggest that elevated ASCT2 expression in CA1 and CA3 in the hippocampus may be associated with the development of depression-like behavior induced by CSDS.

### ASCT2 downregulation in the hippocampus alleviates depression-like behavior

**Figure 3:**
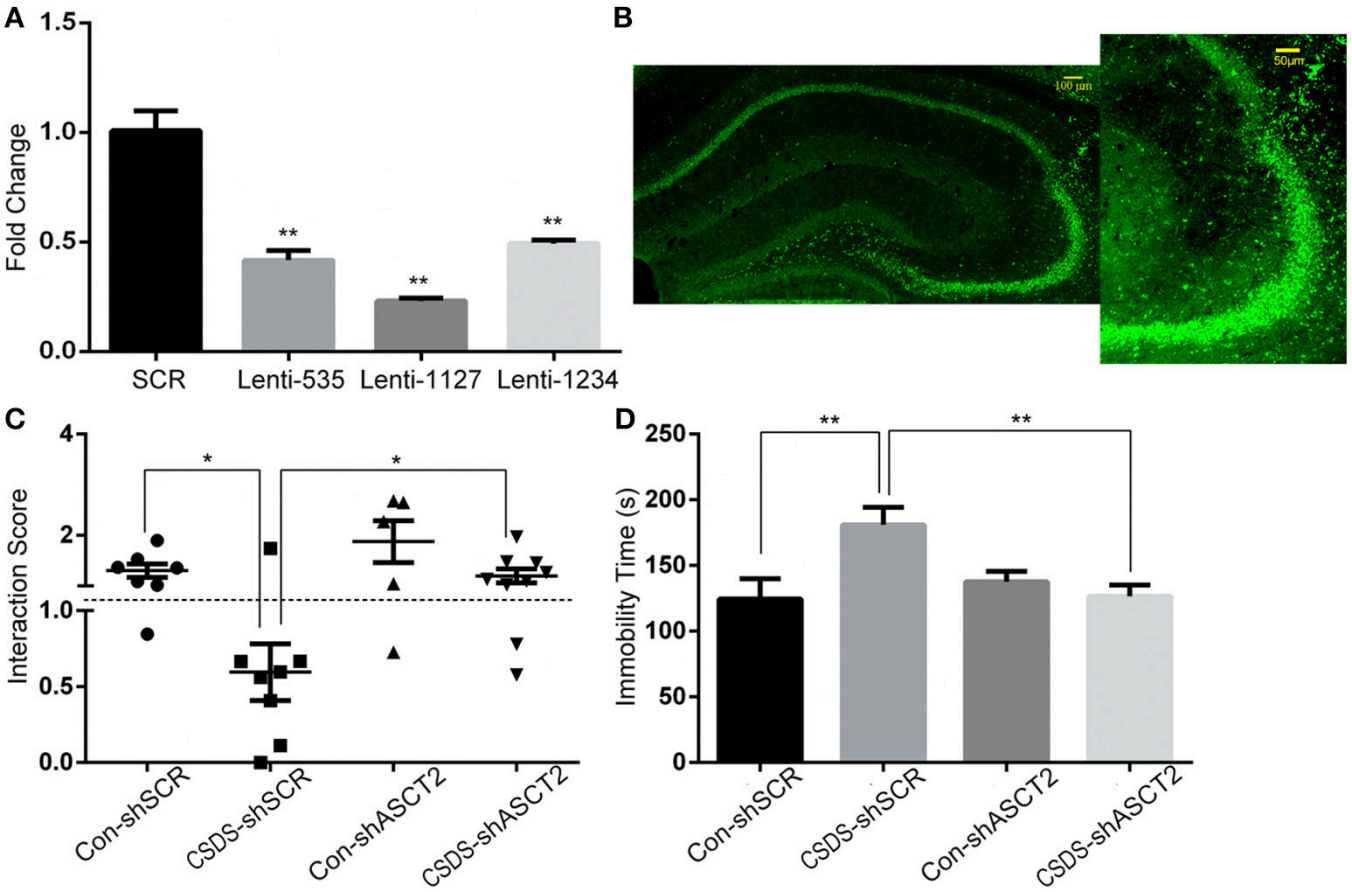
**ASCT2 downregulation in the hippocampus alleviated depression-like behavior in CSDS mice. (A)** mRNA expression of *Asct2* after being infected by lentiviruses carrying *Asct2*-specific (Lenti-535, Lenti-1127, and Lenti-1234) (shASCT2) or scrambled (shSCR) GFP-labeled shRNA, in HT22 hippocampal cells (*n* = 3, ^**^p < 0.01, shASCT2 vs. shSCR; unpaired *t*-test). **(B)** Infected region of shASCT2-1127 in the hippocampus. **(C,D)** Effects of shASCT2 on depression-like behavior in Control (Con) and CSDS mice: the social interaction **(C)** and forced swim test **(D)** (*n* = 7–10; ^*^p < 0.05, ^**^p < 0.01; two-way ANOVA).

To study the behavioral consequences of ASCT2 downregulation, we bilaterally injected lentiviral particles carrying Asct2-shRNA or scrambled (SCR)-shRNA into the hippocampus of CSDS and control mice. As shown in Figure 3A, Asct2-shRNA significantly inhibited *Asct2* mRNA expression in HT22 cells [Lenti-535 (−59.1%); Lenti-1127 (−77.8%); Lenti-1234 (−51.4%); unpaired *t*-test, *p* < 0.01], which demonstrated its interfering efficiency (Figure 3B). Based on these results, Lenti-1127 was used in the following viral injection. In CSDS mice, two-way ANOVA showed a significant main effect of stress [*F*_(1, 25)_ = 11.10, *p* < 0.01] and virus infection [*F*_(1, 25)_ = 7.996, *p* < 0.01], but no interaction effect was observed [*F*_(1, 25)_ = 0.005, *p* > 0.05]; *Post-hoc* analysis showed that ASCT2 downregulation in the hippocampus restored social interaction level reduced by CSDS (CSDS-shSCR vs. CSDS-shAsct2, *p* < 0.05; Figure 3C). In the FST, a significant interaction effect [*F*_(1, 30)_ = 7.366, *p* < 0.05] and marginally significant effect of stress [*F*_(1, 30)_ = 3.337, *p* = 0.077] and virus [*F*_(1, 30)_ = 2.743, *p* = 0.108] were observed; *Post-hoc* analysis revealed that CSDS mice injected with Asct2-shRNA showed significant decrease in immobility compared to the SCR-shRNA-injected CSDS group (CSDS-shSCR vs. CSDS-shAsct2, *p* < 0.01; Figure 3D). Together, these results indicated that ASCT2 downregulation can alleviate depression-like behavior induced by CSDS.

### *Asct2* expression is regulated by histone acetylation in the promoter region

**Figure 4:**
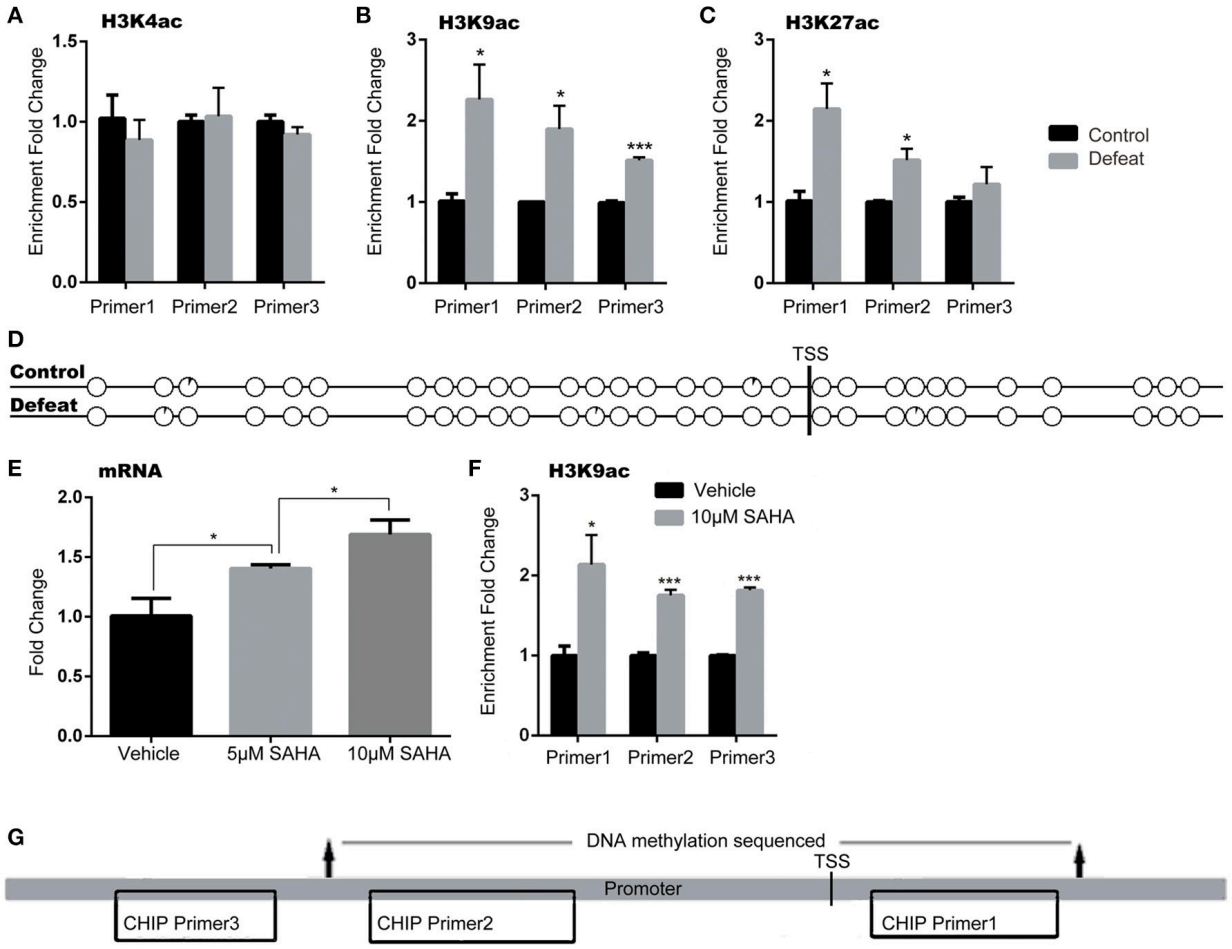
*****Asct2*** mRNA expression was regulated by histone acetylation but not DNA methylation in the promoter. (A–C)** Acetylation of histones H3K4 **(A)**, H3K9 **(B)**, and H3K27 **(C)** in three different regions of the *Asct2* promoter (triplicate in RT-qPCR repeat, ^*^p < 0.05, CSDS vs. control mice; unpaired *t*-test). **(D)** Percentage of methylated clones at different CpG sites in the Asct2 promoter (black in every circle represents the percentage of methylation in each CpG; TSS, transcription start site). **(E)** Relative *Asct2* mRNA expression in HT22 hippocampal cells treated with 5 or 10 mM suberoylanilide hydroxamic acid (SAHA) (triplicate in RT-qPCR repeat, ^*^p < 0.05, one-way ANOVA). **(F)** H3K9 acetylation at the P2 *Asct2* promoter region in SAHA-treated HT22 cells (^*^p < 0.05 and ^***^p < 0.001, 10 mM SAHA vs. vehicle; unpaired *t*-test). **(G)** A diagram shows the locations of the primers used in CHIP assay and sequenced part in bisulfite sequencing.

Accumulating evidence suggests that epigenetic modifications might play a fundamental role in the pathogenesis of stress-related mood disorders (Sun et al., 2012). Here, we tested the levels of DNA methylation and histone acetylation in the *ASCT2* promoter (about 700 bp upstream of the initiation site, according to the Promoter Database; promoter ID: 76201) by bisulfite-PCR sequencing and the CHIP-PCR assay, respectively. The results indicated that acetylation of histones H3K9 and H3K27 but not H3K4 increased in the *Asct2* promoter region in CSDS mice [+125.2% (*p* < 0.05), +89.9% (*p* < 0.05), and +52.5% (*p* < 0.001) for H3K9; +113.2% (*p* < 0.05), +51.5% (*p* < 0.05) and +21.5% (*p* > 0.05) for H3K27; primer 1–3, respectively; triplicate in RT-qPCR repeat; Figures 4A–C]. The DNA methylation level in the *ASCT2* promoter region was very low and no changes were observed (Figure 4D).

To further demonstrate that histone acetylation could regulate *ASCT2* expression, HT22 hippocampal cells were treated with suberoylanilide hydroxamic acid (SAHA), an inhibitor of histone deacetylases (HDACs). SAHA increased *ASCT2* expression in a dose-dependent manner [one-way ANOVA; *F*_(2, 6)_ = 28.05, *p* < 0.001; Figure 4E], which consisted with significant increase in H3K9 acetylation in the *ASCT2* promoter (+113.8%, *p* < 0.05 for Primer 1; +75.4%, *p* < 0.01 for Primer 2; +81.6%, *p* < 0.01 for Primer 3; triplicate in RT-qPCR repeat; Figure 4F). These results confirmed that *ASCT2* expression is acetylation-dependent. Figure 4G shows the locations of CHIP primers location and bisulfite sequencing in the promoter region of *ASCT2*.

### D-serine level is regulated by ASCT2

**Figure 5:**
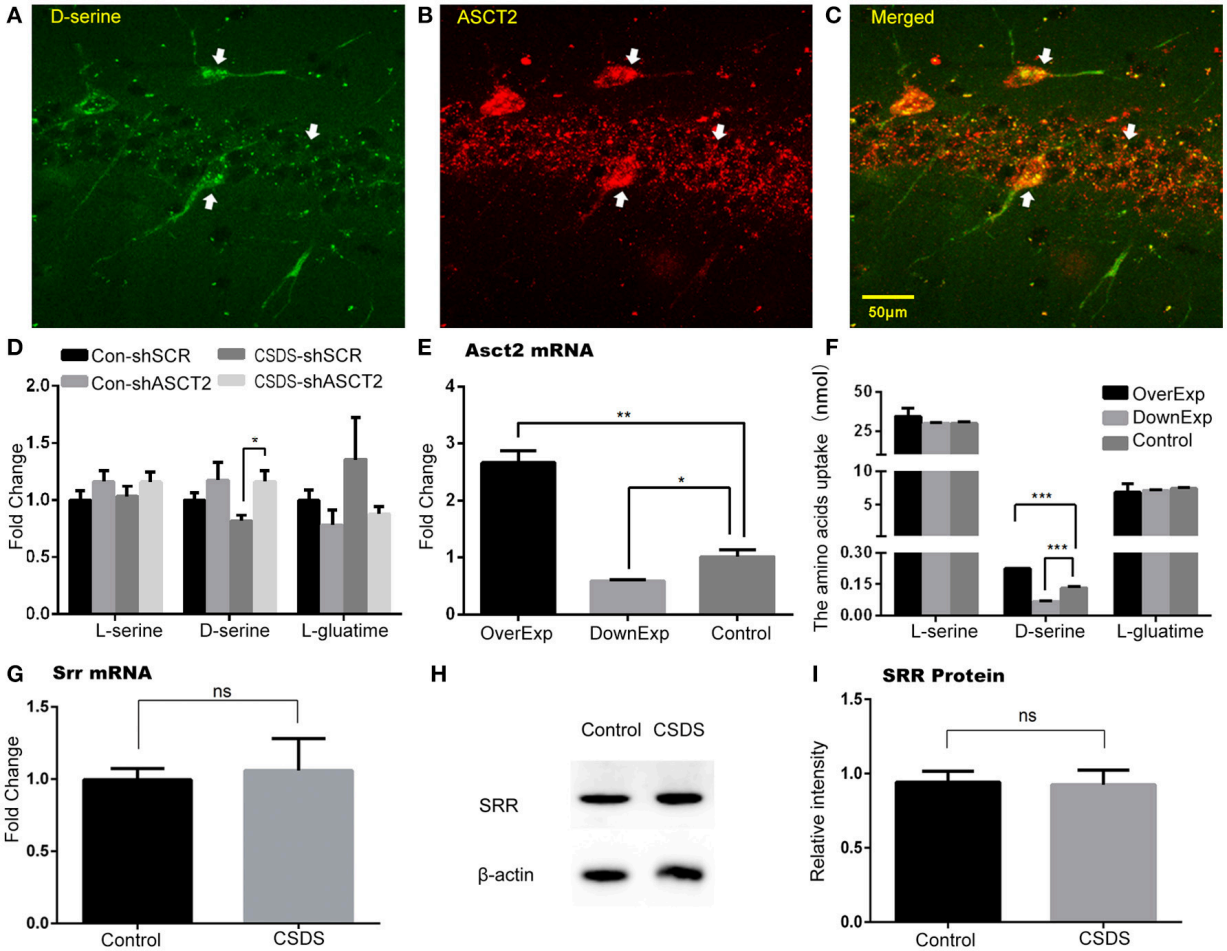
**D-serine level in the hippocampus was regulated by ASCT2. (A–C)** Co-localization of D-serine and ASCT2 in the hippocampus: D-serine **(A)**; ASCT2 **(B)**; Merge **(C)**. **(D)** Relative levels of L-serine, D-serine, and L-glutamine in the hippocampus of control (Con) and CSDS mice infected by lentiviruses carrying *Asct2*-specific shRNA (shASCT2) or scrambled shRNA (shSRC) (*n* = 4, ^*^p < 0.05, two-way ANOVA). **(E)** mRNA expression of Asct2 in HT22 cells after being infected by lentiviruses carrying ASCT2, shASCT2, or shSRC [triplicate in RT-qPCR repeat, ^**^p < 0.01, control vs. over-expressed (OverExp); ^*^p < 0.05, control vs. down-expressed (DownExp); unpaired *t*-test]. **(F)** Cell uptake of L-serine, D-serine, and L-glutamine after being infected by lentiviruses carrying ASCT2, shASCT2, or shSRC. (*n* = 3, ^***^p < 0.0001, control vs. OverExp and control vs. DownExp; unpaired *t*-test). **(G–I)** SRR expression unchanged after CSDS. Relative *Srr* mRNA expression (*n* = 5; unpaired *t*-test) **(G)**; Representative immunoblot **(H)**; quantification of Srr protein expression (*n* = 8, unpaired *t*-test) **(I)**.

Decrease in D-serine concurrently with increase in ASCT2 in the hippocampus of CSDS mice suggested that ASCT2 may negatively regulate D-serine levels in the hippocampus. Immunohistochemistry analysis of hippocampal sections showed that D-serine co-localized with ASCT2 (Figures 5A–C), suggesting a functional link between ASCT2 and D-serine in the hippocampus. Furthermore, two-way ANOVA showed a significant main effect of virus [*F*_(1, 12)_ = 7.25, *p* < 0.05] on hippocampal CA3 region's D-serine; *post-hoc* analysis revealed that shRNA-mediated ASCT2 downregulation restored D-serine level reduced by CSDS (CSDS-shSCR vs. CSDS-shAsct2, *p* < 0.05; Figure 5D). An *in vitro* experiment showed that *ASCT2* overexpression increased the D-serine uptake in the cell medium, while *ASCT2* downregulation decreased the uptake (*p* < 0.001 compared to SCR-shRNA control; Figures 5E,F). Together, these results suggest ASCT2 regulated the D-serine levels in the hippocampus through the effect of D-serine uptake.

**Figure 6:**
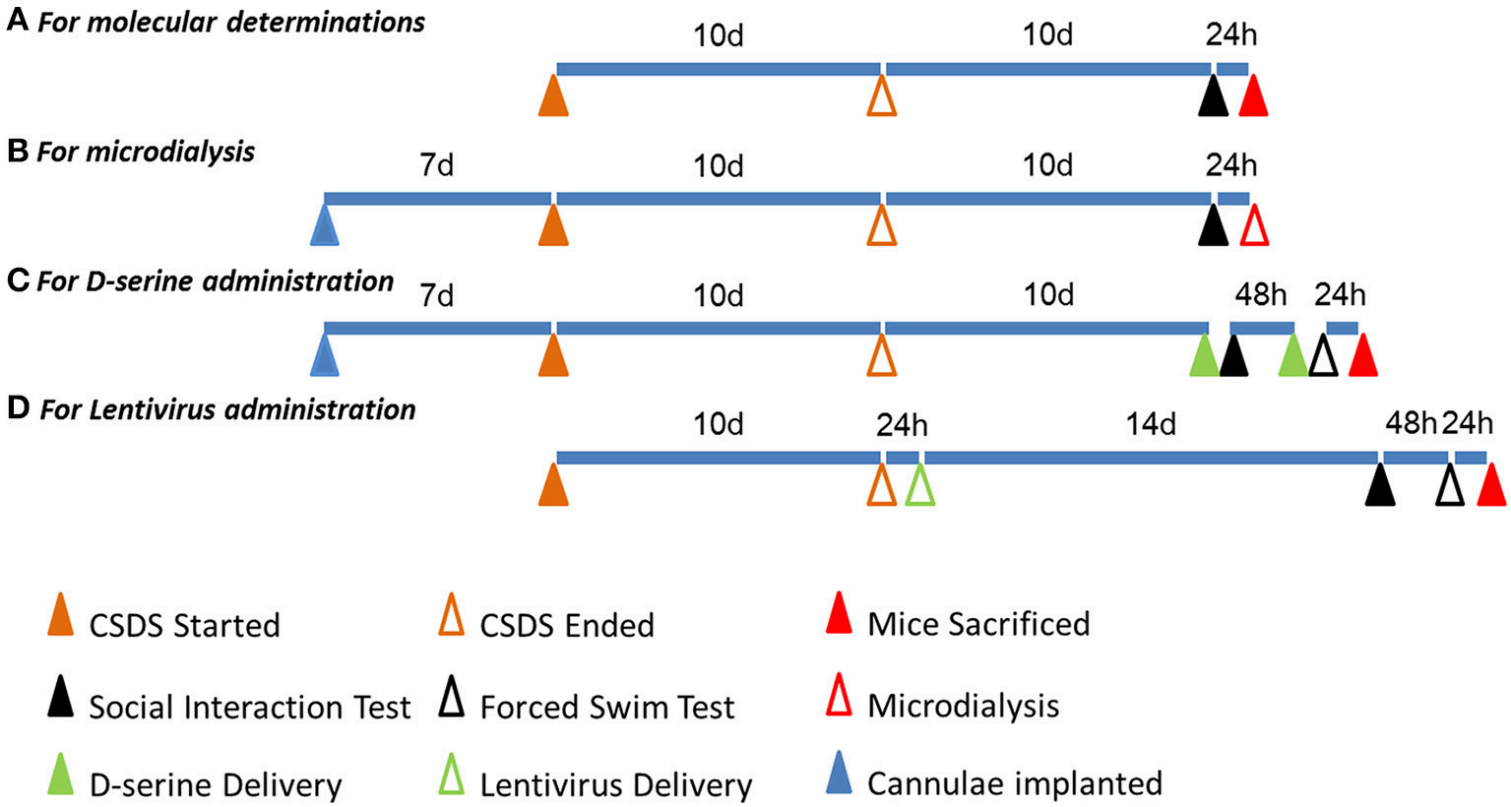
**A timeline diagram of different experiments. (A)** The timeline of molecular determinations: the first, third, fourth, fifth, and eighth batches of mice were manipulated accordingly. **(B)** The timeline of microdialysis: the second batch of mice was manipulated accordingly. **(C)** The timeline of D-serine administration: the sixth batch of mice was manipulated accordingly. **(D)** The timeline of lentivirus administration: the seventh batch of mice was manipulated accordingly.

To exclude the potential role of SRR and DAAO in CSDS, we determined their expression in the hippocampus of CSDS and control mice. Our results showed that there was no difference in SRR mRNA and protein expression between CSDS and control mice (Figures 5G–I). DAAO mRNA was rarely detected in the hippocampus; consistent with previous findings that DAAO is primarily expressed in the cerebellum, and is present in the forebrain, including the hippocampus, at low levels (Nishikawa, 2011). Overall, these data revealed a unique *Asct2*-D-serine pathway in the hippocampus, which may be involved in CSDS-related depression (A timeline diagram provided in Figure 6).

## Discussion

As a co-agonist of the NMDA receptors, D-serine modulates synaptic activity and plasticity; however, its antidepressant effects have been demonstrated only recently in rodents using the FST and learned helplessness paradigms (Malkesman et al., 2012; Otte et al., 2013). Our results indicated rapid and transient antidepressant activity of exogenous D-serine in the hippocampus, manifested as significantly alleviated social avoidance behavior and decreased immobility in the FST; indicating that D-serine levels correlate with the response to chronic social stress and confirming that the hippocampus is a target brain region where D-serine exerts anti-depressive-related effects. Although the underlying mechanisms remain unclear, it is well-documented that D-serine is involved in the induction of long-term potentiation (LTP) in various brain regions, including the nucleus accumbens (Curcio et al., 2013), supraoptic nucleus (Panatier et al., 2006), and hippocampus (Henneberger et al., 2010). In addition, reduced D-serine deceased EPSP and prevent the induction of LTP (Panatier et al., 2006; Henneberger et al., 2010) is consistent with the long-term effects of social stress in rats (Buwalda et al., 2005). These data indicate that CSDS may alter D-serine metabolism in the hippocampus, resulting in reduced D-serine levels and impairment of the NMDA receptor-dependent LTP.

In our study, a significant increase in ASCT2 expression in the hippocampus, especially in the CA1 and CA3 regions, followed CSDS; whereas ASCT2 knockdown alleviated social avoidance and behavioral despair induced by CSDS, demonstrating the direct contribution of hippocampal ASCT2 to depression-like behavior. Moreover, the reduction of ASCT2 expression or inhibition of ASCT2 function increased hippocampal D-serine levels, which, together with the co-localization of ASCT2 and D-serine in the hippocampus, indicate a functional relationship between ASCT2 and D-serine. Furthermore, we showed that downregulation or overexpression of *ASCT2* decreased or increased the uptake of intracellular D-serine *in vitro*. Given that the expression of SRR and DAAO, enzymes also involved in D-serine metabolism, was not affected by CSDS, our results suggest that ASCT2-mediated uptake of D-serine in the hippocampus plays a significant role in the response to CSDS.

In cortical neuron cultures, D-serine uptake kinetics was consistent with ASCT2-mediated transport (Shao et al., 2009), and in C6 glioma cells, a significant correlation between D-serine uptake and *ASCT2* mRNA expression was observed (Sikka et al., 2010), suggesting that ASCT2 may mediate D-serine uptake and be potentially involved in the regulation of D-serine levels in the CNS. Our *in vitro* study revealed that overexpression of *ASCT2* increased uptake of extracellular D-serine. However, the increase in intracellular D-serine was one-tenth of the D-serine uptake after *ASCT2* overexpression (data not shown), indicating intracellular metabolism of D-serine after uptake. Thus, the decrease in extracellular and total D-serine in the hippocampus after CSDS may result from increased uptake of D-serine by ASCT2 activity followed by intracellular metabolism. Since DAAO has a low expression in the hippocampus, we speculate that ASCT2 activation induced by chronic social stress may result in D-serine uptake, followed by intracellular D-serine degradation via alpha, beta-elimination activity of SRR (Foltyn et al., 2005).

Epigenetic mechanisms, such as DNA methylation and histone modification, regulate gene expression and can trigger the development of behavioral responses to stress and antidepressants (Sun et al., 2012). In the present study, we found that CSDS promoted acetylation of histones H3K9 and H3K27 in the *ASCT2* promoter, while DNA methylation was unchanged. Furthermore, we found that SAHA, a selective inhibitor of class I and II HDACs, induced H3K9 acetylation and increased ASCT2 expression in cultured hippocampal cells. Previous studies have demonstrated the functional importance of HDACs in animal models of social defeat. For example, chronic stress led to significant downregulation of *HDAC5* mRNA (class II HDAC) in the nucleus accumbens (Renthal et al., 2007); whereas HDAC5 overexpression in the hippocampus blocked the ability of imipramine to reverse social avoidance (Tsankova et al., 2006). In addition, SAHA administration directly into the mouse nucleus accumbens alleviated social inhibition caused by social defeat stress (Covington et al., 2009). Taken together, these findings support the notion that acetylation-mediated activation of the *ASCT2* promoter is a key mechanism contributing to depression-like behavior induced by CSDS.

In summary, the current study showed that chronic social stress activates *ASCT2* transcription via histone acetylation in its promoter, which decreases D-serine level through elevation of D-serine uptake, resulting in depression-like behaviors. The modulation of D-serine concentration is already considered a promising therapeutic approach to treat disorders associated with altered NMDA receptor activity (Pernot et al., 2012); however, it must be administered at high dosage (grams) to significantly elevate its levels in the CNS (Javitt, 2004) owing to limited diffusion through the blood-brain barrier (Pernot et al., 2012). To this end, a recent study showed that high dose (2.1 g/d) of D-serine reduced feelings of anxiety and sadness and improved cognitive scores in healthy volunteers (Levin et al., 2015). Since potential nephrotoxicity of high D-serine doses has been reported (Orozco-Ibarra et al., 2007), administration of D-serine for treating depression should be further assessed. On another hand, targeting transporters and enzymes involved in the synthesis and metabolism of D-serine may be an alternative treatment approach (Fuchs et al., 2011). Our results indicate that ASCT2 is a potential target for further development of NMDA receptor-based treatments for specific psychiatric disorders owing to its role in the regulation of synaptic D-serine concentrations.

There were several limitations in our study. We only investigated the anti-depressive effects of D-serine and ASCT2 in the hippocampus. D-serine is generally involved in the induction of long-term potentiation in the nucleus accumbens and supraoptic nucleus; and ASCT2 is widespread in mouse brain regions including the cortex, striatum, hippocampus, and cerebellum (Gliddon et al., 2009). Therefore, apart from the hippocampus, their anti-depressive effects in other areas should be investigated. The modulation of the NMDAR is considered a potential therapeutic strategy for major depression. However, both NMDAR antagonists (e.g., ketamine) and agonists (e.g., D-serine) have therapeutic action in depression. One explanation is that both NMDAR inhibition and stimulation converge on the activation of BDNF/mTOR signaling, indicating both receptor antagonists and agonists may be enlisted in treating depression (Chan et al., 2016). Further, studies are needed to examine this hypothesis. In addition, more studies are required to investigate whether patients with depression can be divided into agonist and/or antagonist responders and non-responders.

## Author contributions

Contributed reagents/materials/analysis tools and conceived and designed the experiment: MZ and ZS; Performed the experiment: JW, KZ, and XC; Analyzed the data: JW, XL, and HT; Wrote and revised the manuscript: JW, MZ, and ZS.

## Funding

This study was supported by a grant from the Key Project of the National 12th Five-Year Research Program of China (2012BAI03B02), Special Research Program of the National Health and Family Planning Commission of China (201302002), the AstraZeneca Innovation Centre in China, and the International S&T Cooperation Program of China (2011DFA30670).

### Conflict of interest statement

The authors declare that the research was conducted in the absence of any commercial or financial relationships that could be construed as a potential conflict of interest.
